# Chemical-Functional Diversity in Cell-Penetrating Peptides

**DOI:** 10.1371/journal.pone.0071752

**Published:** 2013-08-09

**Authors:** Sofie Stalmans, Evelien Wynendaele, Nathalie Bracke, Bert Gevaert, Matthias D’Hondt, Kathelijne Peremans, Christian Burvenich, Bart De Spiegeleer

**Affiliations:** 1 Drug Quality and Registration (DruQuaR) Group, Faculty of Pharmaceutical Sciences, Ghent University, Ghent, Belgium; 2 Department of Medical Imaging and Comparative Physiology and Biometrics, Faculty of Veterinary Medicine, Ghent University, Merelbeke, Belgium; University of Edinburgh, United Kingdom

## Abstract

Cell-penetrating peptides (CPPs) are a promising tool to overcome cell membrane barriers. They have already been successfully applied as carriers for several problematic cargoes, like *e.g.* plasmid DNA and (si)RNA, opening doors for new therapeutics. Although several hundreds of CPPs are already described in the literature, only a few commercial applications of CPPs are currently available. Cellular uptake studies of these peptides suffer from inconsistencies in used techniques and other experimental conditions, leading to uncertainties about their uptake mechanisms and structural properties. To clarify the structural characteristics influencing the cell-penetrating properties of peptides, the chemical-functional space of peptides, already investigated for cellular uptake, was explored. For 186 peptides, a new cell-penetrating (CP)-response was proposed, based upon the scattered quantitative results for cellular influx available in the literature. Principal component analysis (PCA) and a quantitative structure-property relationship study (QSPR), using chemo-molecular descriptors and our newly defined CP-response, learned that besides typical well-known properties of CPPs, *i.e.* positive charge and amphipathicity, the shape, structure complexity and the 3D-pattern of constituting atoms influence the cellular uptake capacity of peptides.

## Introduction

Since the discovery about 20 years ago by Frankel and Pabo that the Tat protein of the human immunodeficiency virus (HIV-1) can enter cells [1], cell-penetrating peptides (CPPs) are an increasingly growing part of fundamental and applied biomedical research. Throughout the literature, cell-penetrating peptides are traditionally defined as containing 5–30 amino acids, characterized by a net positive charge, which are able to cross cell barriers without causing significant membrane damage [2]. This property makes CPPs suitable to deliver hydrophilic macromolecules into the cell interior and to the different cellular compartments *in vitro* and *in vivo* [3]. They have already been successfully applied as carriers for problematic cargoes like plasmid DNA, oligonucleotides, short interfering RNA ((si)RNA), peptide-nucleic acids (PNA), proteins and other peptides, small molecules and liposome nanoparticles [4]. This implies that doors have been opened to new efficient peptide drugs [5].

During the last decade, several hundreds of CPPs have already been reported in the literature. In contrast to the traditional definition, CPPs actually present a chemically diverse group of peptides, showing a variety in constituent amino acids and 3D-structure. Three major classes can be distinguished: cationic, amphipathic and hydrophobic CPPs. This structural diversity accounts for the difference in uptake mechanism and level under different conditions between the groups of CPPs. Moreover, coupling the CPP to a cargo can also influence the level and mode of uptake into the cell [6]. Only a few structure-activity relationship (SAR) studies have tried to reveal which structural features are crucial for cellular uptake [7]–[16]. Hydrophobic alpha-helical structures seem to be important, as well as the positive charges from basic amino acids, with arginine favoured over lysine. Although equally contributing to the overall charge, the guanidinum group of arginine can donate two hydrogen bonds compared to one by lysine. Other factors apparently influencing cellular uptake are the peptide length and the conformation of the structure, which was demonstrated by the difference in cellular influx for pVEC and his scrambled analogue [2], [17]. The latter showed a reduced uptake into the cell, probably due to the loss of the N-terminal hydrophobic domain [7]. The influence of the peptide length was demonstrated for the SV40 T antigen, which showed an increase in cellular influx by adding a N-terminal sequence [17].

The available SAR studies only cover a limited set out of the diverse group of CPPs. Moreover, some publications show contradictory results [8], [9], possibly due to different experimental set ups. This impedes drawing general conclusions about the structural features important for cellular uptake. Furthermore, the uptake mechanism of the different CPP groups is still under debate. Today, endocytosis (energy dependent) and direct penetration (energy independent) are suggested to be the two major cellular uptake mechanisms. Depending on the experimental conditions, CPPs use two or more different mechanisms [2].

One approach for predicting CPPs is trial and error, which implies identifying sequences of a suitable length and rich in positive charges in a protein structure [18]. Another approach are the Sandberg expanded z-descriptors, used by Hällbrink et al. [19]. They calculated the bulk property values for a training set of known CPPs and known non-penetrating peptides and averaged over the total number of amino acids. The most relevant descriptors were Z_1_, Z_2_ and Z_3_, describing respectively lipophilicity, steric bulk properties and polarity, the latter having the most predictive power. Cell-penetrating properties of new sequences were predicted based on whether their bulk property values fall within preset intervals, derived from the values of the training set. Z-descriptors make it possible to predict cell-penetrating properties *in silico*, but a major disadvantage is that the sum of descriptors is calculated, hereby neglecting the order of the amino acids. Moreover, the Tat peptide was not considered as a CPP by their search criteria [19]. Another way to predict CPPs is data mining, which is based on finding similarity patterns in a large set of (experimental) data [18]. Artificial neural networks have already been used by Karelson and Dobchev to predict CPPs, based on quantitative structure-activity relationship (QSAR) derived features of a training set of about 100 known (non-)penetrating peptides [20]. Sanders et al. used support vector machine (SVM) classifiers, based on primary features derived from the biochemical properties of 111 known CPPs and 34 non-CPPs, to predict cell-penetrating properties [21]. The authors could experimentally confirm the cell-penetrating ability of the SVM classified CPPs. As primary biochemical properties of peptides were used, their classifiers provided insight in the structural requirements for cellular penetration, *e.g.* positional preference for certain amino acids, like positively charged and aromatic residues.

One can conclude that, although CPPs have been studied for over 20 years, a lot of structural and mechanistic properties still need to be unravelled. Furthermore, it is obvious that the variety of techniques and experimental conditions used to quantify the cellular uptake of CPPs, impedes to directly compare their extent of uptake. Together with the fact that the different CPPs differ structurally and mechanistically, controversies about the uptake mechanisms and artifactual results in the past [22], make it difficult to predict whether a peptide is cell-penetrating or not.

In this article, we explored the chemical space of a set of 186 peptides, for which quantitative data for cellular uptake are available, by use of chemo-molecular descriptors, which numerically express the peptide structure. In addition, we defined a new cell-penetrating (CP)-response, in order to compare the cell-penetrating properties of these peptides in a one-merit figure. This CP-response allows the use and comparison of experimental data obtained with a different experimental set up. By combining the chemical descriptors and the CP-responses, biomolecular modeling and clustering of peptides was performed. Our results confirm already described determining features for cellular uptake, but also provide new insights in structural requirements for cellular uptake of peptides.

## Methods

### Data

Articles describing the uptake of CPPs covering the last five years (2007– March 2012), were gathered using the search engines Web of Knowledge, Google and PubMed. The terms ‘cell penetrating peptides’, ‘uptake cell penetrating peptides’, ‘protein transduction domain’ each separately, as well as ‘cellular uptake’, ‘characterization’, ‘kinetics’, ‘quantification cellular uptake’ and ‘studying uptake’, using the Boolean operator ‘AND’ were used. Specific names of known cell-penetrating peptides (*e.g.* penetratin) were also included as search terms. More publications were obtained by searching in the reference list of suitable articles and reviews. This resulted in publications dating before 2007 (1998–2006). Only those were withheld, where the experimental set up was correct, *i.e.* use of non-fixed cells and removing or quenching of extracellular bound peptide [22]. Moreover, the publications should contain quantitative data or graphs expressing the cellular uptake of CPPs. When no quantitative data were explicitly mentioned in the text, these data were deduced from the available graphs.

### Calculating Chemo-molecular Descriptors

Before the chemo-molecular descriptors of the 186 selected peptides could be calculated, the MM^+^ *in vacuo* optimized structure of the peptides (not amidated), representing the most fundamental peptide structure, was drawn and optimized using HyperChem 8.0 (Hypercube, Gainesville, FL, USA). The geometry optimization was obtained by the molecular mechanics force field method using the Polak–Ribière conjugate gradient algorithm with a root mean square (RMS) gradient of 0.1 kcal/(Å×mol) as stop criterion. Afterwards, these Cartesian coordinate matrices were used to calculate more than 3000 descriptors, using Dragon 5.5 (Talete, Milan, Italy), HyperChem 8.0 and MarvinSketch 5.10.3 (ChemAxon, Budapest, Hungary) software programs. The specific peptide descriptor LogSum_AA_, introduced by our research group, was also included in the descriptor set [23]. The non-discriminative descriptors, *i.e.* constant for all peptides, and one of two highly correlated descriptors, calculated using the Pearson correlation coefficient (absolute correlation >0.95), were eliminated, resulting in a final 186×454 data-matrix for the original descriptors. When all descriptors were divided by the molecular weight, a data matrix of 186×416 was obtained. Next, the data were transformed by z-scaling, ensuring equal contribution of each descriptor to the resulting model [24].

### Multivariate Data-analysis

Multivariate data-analyses were performed using Principal Component Analysis (PCA) and Hierarchical Cluster Analysis (HCA) with SIMCA-P+12.0.0.0 (Umetrics AB, Umeå, Sweden) and SPSS Statistics 20.0.0 (IBM Corp., Armonk, NY, USA) software programs, respectively. Average-linkage HCA clustering was performed using the Euclidean distance as the dissimilarity criterion. After a first PCA-analysis of the dataset, feature selection was performed by selecting the descriptors having a predicted variation value of more than 0.30, resulting in a 186×248 data matrix. For the descriptor set divided by the molecular weight, a 186×210 matrix was obtained.

Multiple Linear Regression (MLR) analysis of the chemo-molecular descriptors, using SPSS Statistics 20.0.0, was performed to build a predictive model for cellular uptake of CPPs. The stepwise method was performed during the MLR process to identify the most significant descriptors using the following criteria: probability of F to enter ≤0.05 and probability of F to remove ≥0.10. After eliminating 12 outliers identified by the Grubbs outlier test (α = 0.05), the CP-responses of 174 peptides were used to build the model (information about the outliers see Table S2).

### Statistics

All statistical analyses of the data were performed using SPSS Statistics 20.0.0 software. Throughout this article, the median of datasets was used as the best measure for central tendency for not normally distributed data.

## Results

### Data

Studies were selected when using protocols, including use of non-fixed cells and removing or quenching of extracellular bound peptide according to Richard et al. [22]. Only pure peptides, not coupled to cargoes or to fatty acid chains, were withheld for this study. At last, we selected only those peptides for which standardizing to the cellular influx of penetratin was possible, allowing to calculate the CP-response for cellular uptake. Finally, a dataset of 186 peptides was obtained, showing high to no or (very) low cellular uptake [7], [9], [11]–[13], [16], [17], [25]–[69] (see Table S1).

The different studies showed a remarkable variety in used techniques and operational parameters to test cellular uptake (Table 1). Inherent to the different techniques used, the protocols of the experiments varied between research groups. This may explain the inconsistent cellular uptake results for some CPPs in the literature, like Tat 48–60, which normally demonstrates a cellular uptake within the same range as penetratin and R9, but was not in reference [17]. The model amphipathic peptide (MAP) showed an unusual low uptake in the study of Wada et al., which is explained by the cell-specific uptake of this CPP [52].

**Table 1:** Experimental differences between studies for cellular uptake of peptides.

Operational parameter	Examples					
Technique	Spectrofluorometry		MALDI-TOF MS		Confocal laser scanning microscopy (CLSM)	
	RP-HPLC		Flow cytometry (FACS)		Atomic Absorption Spectrometry	
	Scintillometry		Splice correction assay		Quantitative image analysis of CLSM images	
	Fluorescence microscopy		–		–	
Positive control	No		Tat 48–60		Transportan 10	
	Penetratin		Tat 47–57		Transportan	
	MAP		R9		YGR6	
	pVEC		D-R9		R8	
Negative control	No		Dextran		Perforin	
	No peptide used		YDEGE		STRRSAMAPR	
	Green fluorescent peptide		YDEEGGG		APRTPGGRR	
Units of quantitative data	µM or nM		pmol or nmol/mg cell protein		SI/mg cell protein	
	ng/mg cell protein		a.u.		Fold change in GeoMean fluorescence	
	Mean fluorescence intensity		RLU/mg		Mean fluorescence intensity/mg cell protein	
	Fold/basal fluorescence		Relative fluorescence intensity		Relative cellular uptake (to control)	
	% of total peptide		% of added peptide		% cellular uptake	
	Cellular fluorescence		Fold change in FITC medium		–	
Label	FITC		5,6-carboxyfluorescein		2-aminobenzoic acid	
	Biotin		Deuterium		Rhodamine	
	NBD		TAMRA		Alexa 488	
	^Ga^DOTA		Texas Red		^125^I	
Cell line	AEC	BMC	HaCaT	HEK293	MC57	*S. cerevisiae*
	HBCEC	CHO (−K1)	Caco-2	HL60	A549	*C. albicans*
	bEnd	U2OS	Cos-7	MDCK	A431	*E. coli*
	MCF-7	Jurkat	MOLT-4	HeLa	Hela pLuc705	*B. megaterium*
	NIH-3T3	RAW264.7	BA/F3	K562	BT-20	N2a
	KB	RAW	U373 MG	Daudi	Sf9	MDA-MB-231
	HT-29	SKMel37	DAMI	A549	U251	KG1a
	TF-1	ESC	NC	Sca-1^+^Lin^−^	HEK293	L929
	Calu-3	MDA	HER	TM12	CCRF-CEM	–
Incubation concentration	10 nM	200 nM	0.1 µM	0.33 µM	0.4 µM	0.8 µM
	1 µM	1.8 µM	2 µM	2.5 µM	3 µM	3.1 µM
	3.5 µM	4 µM	4.5 µM	5 µM	6 µM	6.3 µM
	7.5 µM	10 µM	12.5 µM	15 µM	20 µM	25 µM
	30 µM	40 µM	50 µM	100 µM	110 µM	200 µM
	400 µM	800 µM	1.6 mM	–	–	–

### Defining a Cell-penetrating Response

Because of the variety in experimental settings throughout the literature, the cellular uptake results of the available CPPs are difficult to directly compare and are expressed using different units, as listed in Table 1. Therefore, a cell-penetrating (CP)-response, a unified response expressing the cellular uptake efficiency of CPPs, would be of great help to obtain a clear overview over the cellular influx capacities of the CPPs described in the literature.

Penetratin, one of the first discovered CPPs and often described in the literature, is the most used positive control in uptake studies of other peptides. Therefore, penetratin was considered as a general positive control and used to normalize the responses for cellular uptake. Before a CP-response could be defined, several assumptions were made: *(1) cell and label differences were neglected.* As shown in Table 1, about 50 different cell lines and 12 different labels were used. The different nature of the labels was not considered when chemically defining the peptide structure. *(2) The uptake of the negative control was considered to be negligible*. *(3) The maximal values of cellular uptake during an experiment were used to cope with a possible time effect. (4) If a positive control was used in a study, it was considered as an internal standard* and could be used to average variations in operational parameters. Finally, *(5) a linear correlation between the extracellular and intracellular peptide concentration* was assumed, although it cannot be excluded that there is a specific concentration effect [37], [39], [41], [42], [60]. This last assumption was necessary, because to calculate the CP-response, the quantitative value for cellular uptake was first corrected for the incubation (extracellular) concentration resulting in a concentration normalized response. Then, the latter response was normalized to the positive control penetratin, according to the following equation:
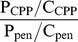
(1)where P_CPP_/C_CPP_ and P_pen_/C_pen_ are the concentration normalized influx responses for a CPP and penetratin respectively in the same study.

As already mentioned before, not all studies included penetratin as a positive control. When another positive control than penetratin was used, the median of all available ratios of that alternative positive control over penetratin was used to normalize the response to penetratin:
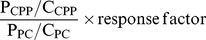
(2)where P_CPP_/C_CPP_ is the concentration normalized influx response for a CPP, P_PC_/C_PC_ for a positive control in the same study different from penetratin and the response factor is the median of all ratios of the concentration normalized responses of the positive control over the concentration normalized responses of penetratin, as expressed in formula *(1)* (Table 2).

**Table 2:** Overview of the used positive controls in studies for cellular uptake of peptides and their CP-response.

Positive control	CP-response
MAP	2.05
Penetratin	1.00
pVEC	1.31
R9	1.00
Tat 47–57	0.31
Tat 48–60	0.22
Transportan 10	1.64

A third possibility was that no positive control was used in the cellular uptake study. Then, the CP- response was calculated using the following equation:
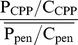
(3)with P_CPP_/C_CPP_ being the concentration normalized influx response for a CPP and 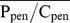 the median of all concentration normalized influx responses of penetratin, obtained using the same technique as the considered influx response (*i.e.* having the same unit).

**Figure 1:**
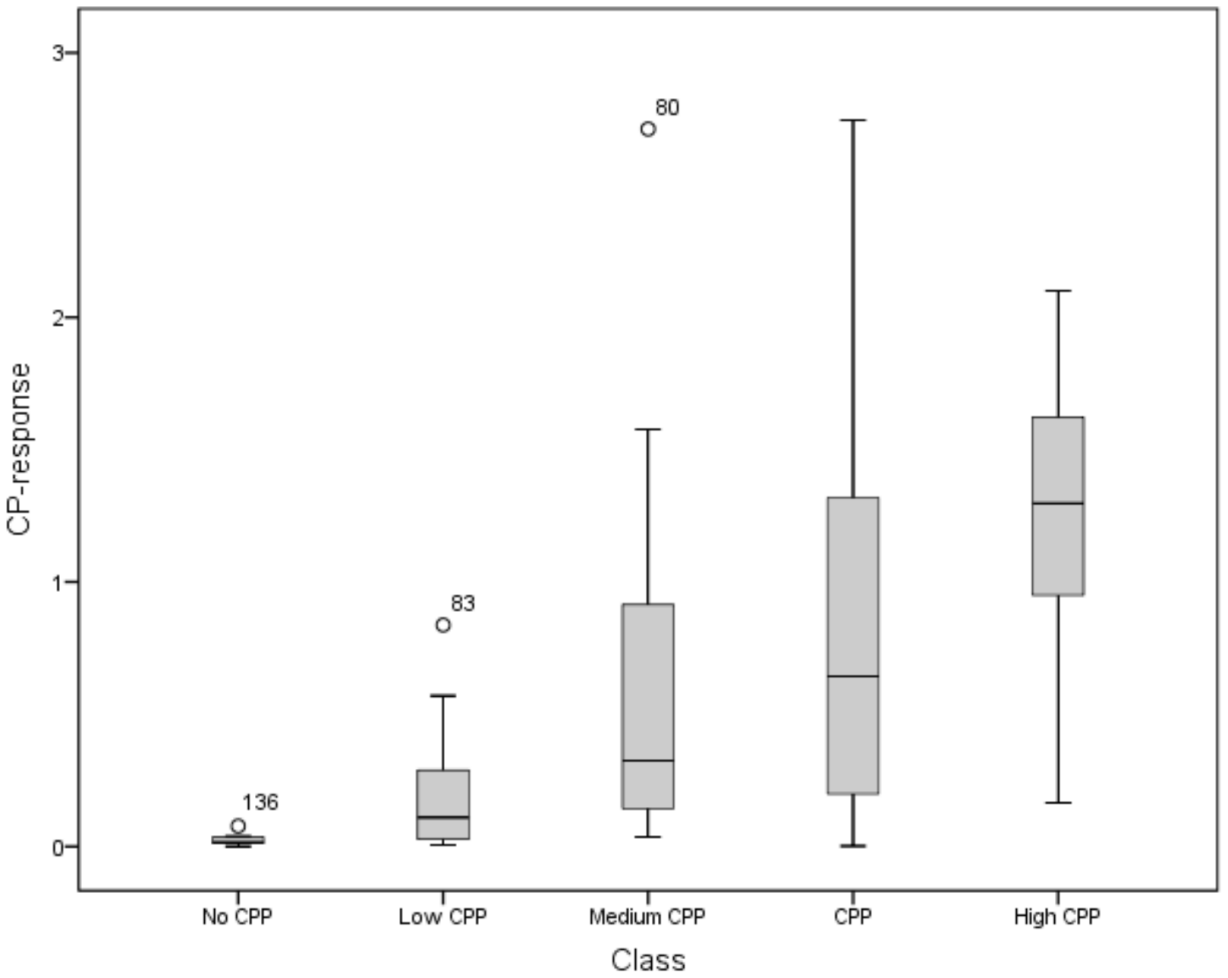
Distribution of the CP-responses in five different CPP classes as defined by the authors.

If more than one CP-response was available for a peptide, the median CP-response was calculated. Over all peptides, the CP-response ranged from 0.001378 to 2.744. The ranking of the peptides based on their CP-response, roughly corresponded with those found in the literature, *e.g.* the CP-response increased as follows: Tat 48–60< R9 ≈ penetratin <pVEC<transportan 10< MAP< transportan. This was in agreement with the overall study conclusions: Tat 48–60 mostly showed the lowest cellular influx [17], [26], [30], [31], [33], [34], [38], followed by R9 and penetratin [17], [25], [26], [28], [30]–[34], [38]. The peptides pVEC, transportan 10, MAP and transportan showed higher cellular influx than Tat 48–60, penetratin and R9. Transportan mostly showed a higher cellular influx than transportan 10 [10], [28]. Moreover, as a proof of concept, we investigated all manuscripts providing the quantitative data for cellular influx for the 186 peptides and compiled for each peptide how the authors estimated (subjectively) their cell-penetrating properties (see Table S3). We identified *five classes*: *no CPP*, *low CPP* (described as low CPP, low efficient, low effective, slow, nearly unmeasurable), *medium CPP* (described as medium CPP, efficient, effective) and *high CPP* (described as high CPP, highly, extremely effective, extremely efficient, rapid). When the authors only described the peptide as cell-penetrating, without any scaling or subjective ranking, these peptides were classified as *CPP*. Next, the distribution of the CP-responses in the five different classes was evaluated using Box-Whisker plots (see Figure 1). The median CP-response increased over the different classes from no CPP over low CPP, medium CPP and CPP to high CPP, indicating that peptides having a high or low calculated CP-response were also estimated in the same way by the researchers. This more detailed analysis thus demonstrated that the CP-response is indicative for the extent of cell-penetration of a peptide.

### Exploration of the Chemical Space of CPPs

**Figure 2:**
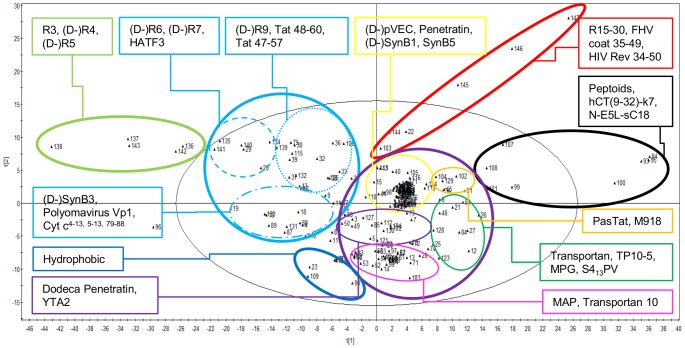
Score plot of the first versus the second principal component of the PCA-analysis of 186 peptides. The six main clusters of peptides are indicated by a bold line (light green, light blue, red, purple, black and dark blue clusters), while the eight subclusters are encircled by a thin line (light blue dashed and/or dotted line, yellow, orange, dark green, purple and pink clusters). For each cluster, some examples of peptides are indicated.

To determine the chemical space of a set of 186 peptides, which were investigated for cell-penetrating properties, a PCA and HCA-analysis of their calculated descriptors was performed. The first two principal components (PCs) of the calculated PCA-model explained already 62.6% of the total variability (Table 3). Based on the dendrogram of the HCA-analysis and the score plot of the first two PCs of the PCA-analysis, the 186 peptides could be categorized into six main clusters, which could be subdivided into eight subclusters (Figure 2).

**Table 3:** Summary of the PCA-analysis of the original descriptors, describing the eigenvalues of the covariance matrix, the total variance explained (cumulative R^2^) and the predictive ability (cumulative Q^2^).

Principal Component	Eigenvalue	Cumulative R^2^	Cumulative Q^2^
1	86.9	0.467	0.448
2	29.5	0.626	0.602
3	12.1	0.691	0.639
4	11.6	0.753	0.701
5	5.74	0.784	0.720
6	5.16	0.812	0.743
7	4.42	0.836	0.764
8	3.53	0.854	0.781
9	2.58	0.868	0.789
10	2.17	0.880	0.797
11	1.94	0.890	0.807

The loading plot indicated that the first principle component (PC1) is mainly influenced by the mass, shape and connectivity of the peptides, while the second principle component (PC2) was determined by hydrophilicity and lipophilicity. In Figure 2, the peptides with high molecular weight (MW), surface area, molecular volume and number of hydrogen acceptor atoms were situated on the right along the horizontal axis and inherently these peptides had a higher number of peptide bonds (represented by the descriptors nRCONHR and C-040). The peptides on the right were also characterized by a more voluminous, complex and less compact structure. On the other side of the horizontal axis, the smaller, more symmetrical and compact peptides were located. On the PC2-axis, peptides mainly consisting of hydrophilic amino acids, like the basic arginine and lysine residues, represented by the high pI values of these peptides, were situated at the top. When descending to the bottom, the peptides turn more hydrophobic, indicated by higher log P values, hydration energy and BLI values (Kier benzene likeliness index), the latter describing the extent of molecular aromaticity.

The light green cluster at the left in the score plot represented short oligo-arginines (R3–R5), showing a very low median CP-response of 0.0769. The light blue subclusters contained cationic peptides, which differed in charge and peptide length (increasing from the left to the right). The light blue dashed-dotted subcluster (*e.g.* SynB3 and polyomavirus Vp1), showed a low median CP-response of 0.0392, while the dashed (*e.g.* R6, R7 and HATF3) and dotted (*e.g.* R9 and Tat 48–60) light blue subclusters had a mediocre cellular influx with median CP-responses of 0.323 and 0.464, respectively. The yellow and orange subclusters, which were centrally located in the PCA score plot, formed mixed clusters, as they contained both cationic and amphipathic peptides. The pink and purple amphipathic subclusters had median CP-responses of 0.181 and 0.302, respectively. The yellow subcluster (*e.g.* pVEC and penetratin), orange subcluster (*e.g.* PasTat and M918) and the dark green subcluster (*e.g.* transportan and MPG) had the highest values for the median CP-response, ranging from 0.511 to 0.729 and 0.798, respectively. These peptides were cationic and/or amphipathic and are composed of 15–27 amino acids. Remarkably, the group of peptides, showing a high CP-response could be subdivided in two groups: those having a positive PC2 value, which were mainly arginine rich (yellow and orange subcluster) and those having a negative PC2 value (dark green subcluster), which were mainly lysine rich. Although it was previously stated that arginine residues are favourable over lysine for cellular influx [2], our data did not confirm this statement. Peptides showing the highest CP-response had a high charge density or show amphipathicity. The latter peptides were centrally located in the score plot and were rich in sulfur-containing residues, especially methionine, as well as in aromatic amino acids.

The hydrophobic peptides, which are alanine, glycine, leucine, proline and valine rich, were located at the bottom of the score plot and showed a mediocre, but significant influx (median CP-response of 0.354). The peptides of the red cluster were highly charged and showed a high CP- response (median of 0.764). The cluster was mainly composed of oligoarginines of more than 15 residues, which are known for their cellular toxicity [12]. The black cluster consisted of voluminous, high molecular weight peptides, *i.a.* some peptoid structures, showing a very low cellular influx (median CP-response of 0.166).

As PC1 was mainly dominated by the molecular weight, the same PCA-analysis was performed, but using all descriptors divided by the molecular weight in order to neutralize its MW size-effect, although some descriptors were already corrected for the MW. However, this modification of the descriptors did not deliver extra information. The calculated PCA-model resulted in similar clusters of CPPs (see Table S4 and Figure S1).

### Functional Diversity of CPPs

Using our newly defined CP-response and the calculated chemo-molecular descriptors of the peptides, a stepwise MLR-model was constructed to predict the cell-penetrating ability of new peptides. Variability in the CP-response, due to the experimental variations as well as to the assumptions made, was also taken into consideration by introducing random response noise ranging between 0.90 and 1.10. With those *in silico* noised responses, covering thus 20% of variability, new datasets were created (MLR1 to MLR10). By performing the MLR-analysis of these datasets (Table 4), the descriptors most robustly influencing the CP-response, *i.e.* descriptors which were withheld in more than half of the MLR-models, were selected. In Table 5, the meaning of these robust descriptors influencing the cell-penetrating properties are listed.

**Table 4:** Overview of the most robust descriptors influencing the CP-responses in the 11 MLR-models.

	MLR	MLR_1_	MLR_2_	MLR_3_	MLR_4_	MLR_5_	MLR_6_	MLR_7_	MLR_8_	MLR_9_	MLR_10_	Mean
R^2^	0.621	0.589	0.493	0.515	0.619	0.617	0.508	0.587	0.525	0.572	0.615	0.569
Adjusted R^2^	0.577	0.545	0.458	0.478	0.578	0.572	0.471	0.542	0.487	0.532	0.567	0.528
**Descriptor**	**Coefficients** 1											**#**
B04[N-N]	0.175	0.285	0.287	0.298	0.228	0.154	0.183	0.251	0.203	0.305	0.187	11
GATS5m	0.401	0.573	0.321	0.298	0.541	0.435	0.389	0.443	0.396	0.612	0.670	11
G2e	−0.184	−0.141	−0.186	−0.221	−0.205	−0.186	−0.218	−0.226	−0.215	−0.177	−0.181	11
nCt	0.465	0.482	0.570	0.547	–	0.453	0.491	0.588	0.555	0.215	–	9
nROR	0.244	0.198	–	–	0.322	0.231	–	–	–	0.300	0.320	6
T(N.S)	0.912	0.461	–	–	0.897	0.940	–	–	–	0.799	0.607	6
G3u	−0.184	−0.137	–	–	−0.224	−0.183	–	–	–	−0.190	–	5
Mp	0.548	0.352	–	–	0.525	0.553	–	0.307	–	–	–	5
Mor15p	−0.656	–	–	–	−0.791	−0.673	–	–	–	−0.366	−0.275	5
Mor26m	−0.319	–	−0.202	−0.209	−0.361	−0.318	–	–	−0.191	−0.305	–	7
GATS7e	–	–	0.922	1.066	–	–	1.127	1.233	1.143	–	–	5
GATS7p	–	–	−0.682	−0.761	–	–	−0.798	−0.944	−0.806	–	–	5
Mor16p	–	−0.316	−0.385	−0.419	–	–	−0.478	−0.482	−0.391	–	−0.301	7
Mor27m	–	–	−0.410	−0.404	–	–	−0.327	−0.298	−0.387	–	–	5
Mor27e	–	–	0.248	0.291	–	–	0.202	0.217	0.274	–	–	5

1 For each model, the coefficients of the significant descriptors are indicated.

**Table 5:** Meanings of the robust descriptors influencing significantly the CP-response of peptides.

Descriptor	Meaning	Class
B04[N-N]	Presence/absence of N-N at topological distance 4	2D binary fingerprints
GATS5m	Geary autocorrelation - lag 5/weighted by atomic masses	2D autocorrelations
G2e	2st component symmetry directional WHIM index/weighted by atomic Sanderson electronegativities	WHIM^1^ descriptors
nCt	Number of total tertiary C(sp^3^)	Functional group counts
nROR	Number of ethers (aliphatic)	Functional group counts
T(N.S)	Sum of topological distances between N.S	Topological descriptors
G3u	3st component symmetry directional WHIM index/unweighted	WHIM descriptors
Mp	Mean atomic polarizability (scaled on Carbon atom)	Constitutional descriptors
Mor15p	3D-MoRSE - signal 15/weighted by atomic polarizabilities	3D-MoRSE^2^ descriptors
Mor26m	3D-MoRSE - signal 26/weighted by atomic masses	3D-MoRSE^2^ descriptors
GATS7e	Geary autocorrelation - lag 7/weighted by atomic Sanderson electronegativities	2D autocorrelations
GATS7p	Geary autocorrelation - lag 7/weighted by atomic polarizabilities	2D autocorrelations
Mor16p	3D-MoRSE - signal 16/weighted by atomic polarizabilities	3D-MoRSE^2^ descriptors
Mor27m	3D-MoRSE - signal 27/weighted by atomic masses	3D-MoRSE^2^ descriptors
Mor27e	3D-MoRSE - signal 27/weighted by atomic Sanderson electronegativities	3D-MoRSE^2^ descriptors

1 Weighted Holistic Invariant Molecular descriptors.

^2^3D-Molecular Representation of Structures based on Electron diffraction.

The descriptor B04[N-N] is a 2D-binary fingerprint descriptor, representing the presence or absence of the specific atom pair N-N at a topological distance of four bonds. Our models indicated that the presence of such a N-N pair has a positive influence on the cell-penetrating response. When looking at the amino acid structures, this N-N bond at topological distance four is found in asparagine and histidine residues. The latter is a weak alpha-helix former and thus may be important to establish the secondary amphipathic structure of peptides [70]. The GATS5m, GATS7p and GATS7e descriptors are Geary 2D-autocorrelation descriptors, which describe the topology of the peptide in association with atomic masses (m), polarizabilities (p) and Sanderson electronegativities (e). At specific path length (lag) five, the atomic masses have a high positive contribution to the cell-penetrating properties, while at lag seven, a positive (weighted by atomic Sanderson electronegativities) or negative (weighted by atomic polarizabilities) influence on our CP-response was observed. GATS7e shows the dispersion of electronegative atoms at a topological distance equal to seven bonds in a peptide, while the value of GATS7p shows the importance of atomic polarizabilities over the same topological distance. Peptides having high (GATS5m and GATS7e) or low (GATS7p) values of these descriptors, were rich in basic amino acids, arginine and lysine, as well as the aromatic amino acid tryptophan.

3D-Molecule Representation of Structures based on Electron diffraction (3D-MoRSE) descriptors are 3D-molecular descriptors derived from scattering transform functions, reflecting various physicochemical properties, like atomic polarizability (signals 15 and 16), atomic masses (signals 26 and 27) and atomic electronegativity (signal 27) [71]. From these 3D-MoRSE descriptors could be derived that the position of these physicochemical properties in the 3D-space is crucial for cell-penetrating properties. Based on these descriptors, a favourable cellular influx was predicted for the amphipathic and/or cationic subclusters of the PCA-analysis, *i.e.* the dark green, pink, purple and yellow subclusters. Moreover, the peptides belonging to the dark green and yellow subclusters showed the highest median CP-response, which was also predicted based on their values of the robust 3D-MoRSE descriptors. 3D-descriptors characterizing the symmetry of the peptides also robustly influenced the CP-response: the symmetry-directional WHIM descriptors G2e (weighted by atomic Sanderson electronegativities) and G3u (unweighted) negatively influenced the cell-penetrating properties, indicating that the cellular influx of peptides increased with decreasing peptide symmetry [71]. Peptides containing branched and hydrophobic amino acids, *e.g.* valine, leucine and isoleucine, as indicated by the descriptor nCt, accounting for the number of tertiary carbon atoms showed a higher CP-response. Also the T(N.S) descriptor referring to the presence of sulfur-containing amino acids, and the mean atomic polarizability (Mp) contributed positively to the cellular penetration. Methionine as well as the hydrophobic amino acids are also (strong) alpha-helix formers and thus important for establishing a secondary amphipathic structure. Finally, the nROR descriptor, which was an unexpected robust descriptor, also positively influenced the CP-response. The cationic amphiphilic polyproline helices (CAPHs) contain such ether functions to link the hydrophobic and hydrophilic residues. Although the MLR-analysis did not directly point to the importance of a positive charge for cellular uptake, the information contained in the robust descriptors indicated its influence as well as of a secondary amphipathic structure.

## Discussion

Studies of the cellular uptake of cell-penetrating peptides demonstrate a great variety in experimental conditions, as illustrated in Table 1. These differences in used techniques and operational parameters, are at least partly responsible for discrepancies in conclusions about the cellular uptake of certain CPPs, like *e.g.* the uptake mechanism. In Table S3, the available information on the mechanism of cellular uptake of our selected peptides is listed. There are three main mechanisms of cellular entry: *(1) direct penetration*, wich can be subdivided into (a) inverted micelle formation, (b) pore formation, (c) carpet-like model, (d) membrane thinning and (e) nucleation zones. The second mechanism is *(2) endocytosis*, with subcategories (a) micropinocytosis, (b) dependent on coat proteins and (c) independent on coat proteins. Some publications also define a third mechanism: *(3) energy-dependent, but not endocytosis* [2], [12], [72], [73]. From Table S3 can be derived that the different studies on the uptake mechanism of CPPs show an inconsistency in cellular uptake mechanism. Cell-penetrating peptides use different mechanisms of entry, either simultaneously or as function of experimental factors, like the extracellular concentration, cell line, presence of a cargo, incubation time and temperature [2], [42], [44].

Clearly, there is an urgent need for harmonization of the experimental conditions in the investigations of cellular uptake of peptides, like other authors have already suggested in the past [18], [20]. Especially, the use of a standard positive control or controls, *e.g*. penetratin, is recommended, as it allows to neutralize to some extent the differences in experimental conditions. Therefore, we defined a CP-response, a unified response which allows the comparison of experimental data of the cellular influx of peptides. Several assumptions were made, which cause, together with the existing experimental variations, some variability in our CP-response. Nevertheless, the hitherto described cell-penetrating peptides can be compared using this CP-response and new conclusions about the structure-activity modeling of these peptides can be drawn.

**Figure 3:**
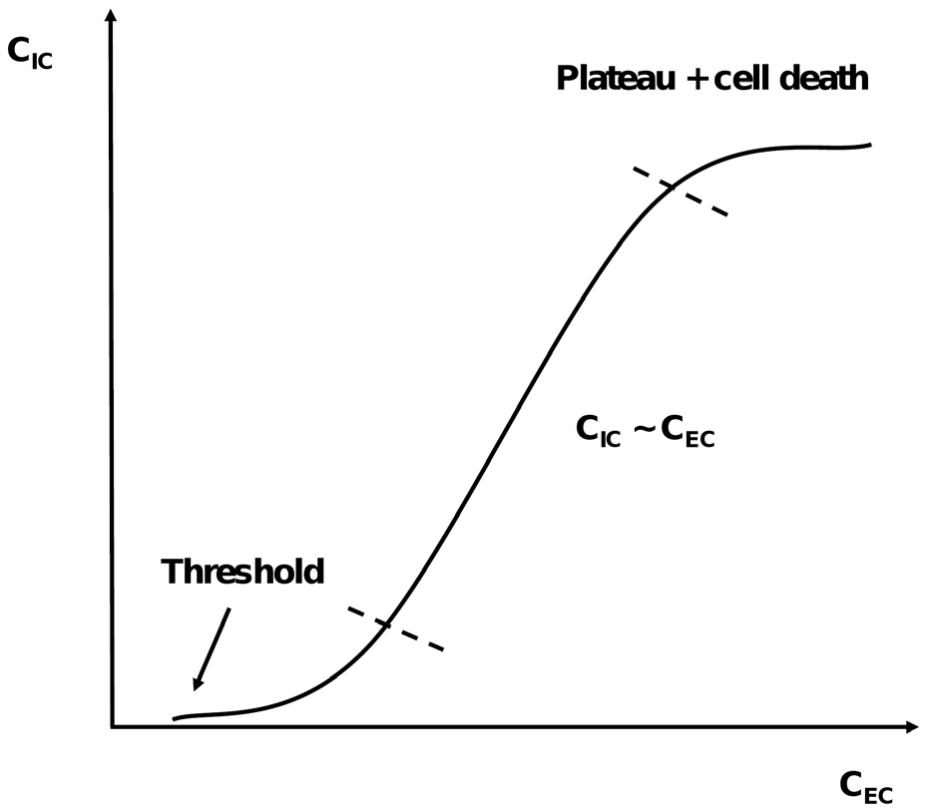
Supposed dependence of the intracellular CPP concentration on the extracellular concentration when performing cellular influx studies.

As a first assumption, cell and label differences were neglected, as a wide range of cell lines and detection labels are used throughout the literature. It is clear that different cell lines have different membrane characteristics, which influence the cell-penetrating properties [17], [22], [25], [27], [30], [32]–[34], [37], [38], [40], [41], [43]–[45], [53], [55], [56], [58], [64]–[67]. We also assumed penetratin as a general positive control, because it is quite often used and is well characterized, being one of the first described CPPs. It was also necessary to correct the uptake responses for the incubation concentration, as there exists a clear relationship between the extracellular and intracellular concentration of CPPs. Therefore, we assumed a simple linear relationship, justified by the fact that only a few studies have already investigated the internalization dependence on the extracellular peptide concentration, not allowing more complex models to be used. For most CPPs, there is indeed a correlation between the intracellular and the extracellular concentration [37], [39], [41], [42], [60]. On the other hand, some peptides, like R9, hLF and Tat 47–57, show a sudden sharp increase in intracellular concentration, when a certain extracellular concentration is reached [41], [42]. Still for other peptides, the extracellular concentration needs to exceed a threshold concentration before cellular uptake takes place. Some authors explain this phenomenon by the fact that the uptake mechanism of CPPs depends on the extracellular concentration [42]. Moreover, Hällbrink et al. [74] showed that the uptake of CPPs may also be dependent on the peptide-to-cell ratio, as demonstrated for MAP and penetratin. Besides, some CPPs show toxic effects starting from a certain extracellular concentration [37], [39]. Taking the above findings in consideration, we visualized the intracellular versus extracellular concentration curve for CPPs as a sigmoid (see Figure 3), characterized by a threshold value for influx, which was for all available peptide data about 1 µM. When the threshold is reached, the intracellular concentration increases in function of the extracellular concentration, followed by flattening of the curve until a plateau value for intracellular concentration is reached, possibly due to cell death. The threshold value for influx is CPP and cell line dependent. For most CPPs however, only one extracellular concentration is investigated, which makes it impossible to reconstruct the full sigmoid curve dependence. We applied a linear model, realizing that this approach is an over-simplification, leading to increased variability and bias. It is clear that studying the correlations between intracellular and extracellular concentration, would give more insights into the uptake mechanisms of the peptides, as well as into the toxicity profile.

Our dataset contained peptides showing very low to high cellular influx (CP-response of 0.001378 to 2.744), indicating that our dataset covered a sufficiently wide range of cell-penetrating responses. Moreover, the ranking of the peptides based on the CP-responses, corresponds roughly with those found in the literature, when considering the most studied and compared CPPs. This indicates that our approach is a valuable quantitative way to assess CPP properties, which was also demonstrated by the evaluation of the distribution of the CP-responses in the five different classes of CPPs as defined by the authors. From Figure 1 can be derived that the medium CP-response increases over the different classes from no CPP to high CPP. Still there exists a clear overlap in CP-responses between the different classes. The lower whiskers of the distribution of the medium CPP, CPP and high CPP classes are extended to almost zero response, indicating that they also contain non- or low-penetrating peptides, according to our proposed CP-response. We evaluated the peptides composing these lowest values and concluded that they can often be explained by an incorrect descriptive conclusion of the authors. Possible reasons are that the classification was based on experiments without trypsinization, while also experiments with trypsinization of the cells were performed, or that much higher incubation concentrations than normally applied are used in order to reach cell-penetration, leading to low CP-responses as they are concentration corrected [17], [51]. Nevertheless, this observed consistency strengthens the value of our CP-response.

The exploration of the chemical space of the 186 peptides, investigated for cell-penetrating properties, confirmed some known features about CPPs, thus supporting our approach, but also revealed some new insights in the structural diversity of these peptides. The molecular weight, surface area, molecular volume, the number of hydrogen bond acceptors, hydrophobicity and charge determined the main clusters in the PCA-analysis. These characteristics join with previous findings about important properties for cellular influx, *i.a.* z-scales used by Hällbrink et al. [19]. However, our PCA-analysis indicated that also the shape and complexity of the structure differ within the group of CPPs. In the score plot of the PCA-analysis (Figure 2), there was a clear trend in symmetry, complexity and compactness of the structure: extremes for these descriptors give low CP-responses for the peptides. From this exploration of the chemical space of CPPs, it can be derived that not only the constituent amino acids determine cell-penetrating properties but also their position. This contrasts the current general opinion that the 3D-structure is not significantly influencing the cellular uptake, except for the secondary amphipathic CPPs [6]. Moreover, our 3D-structures are calculated based on a theoretical phase, *i.e.* MM^+^ *in vacuo* optimized structures according to Hyperchem molecular mechanics, which is independent from its biological medium and interactions.

The light green cluster in Figure 2 consists of oligo-arginines of up to five arginines and shows a very low to negligible CP-response, consistent with the conclusions of Mitchell et al. [12]. On the other hand, based on the characteristics of the clusters with the highest unified response, high density of positive charges and amphipathicity favour cellular uptake. The amphipathic peptides were located centrally in the score plot of the PCA-analysis and were characterized by a high extent of sulfur-containing residues, as well as aromatic amino acids. These features are indeed important for establishing a secondary amphipathic structure. According to Chou and Fasman, methionine and the aromatic amino acids, phenylalanine and tryptophan, are (strong) alpha-helix formers and as hydrophobic amino acids they contribute to hydrophobic interactions when establishing the secondary structure [70].

Although MLR only captures a linear correlation between descriptors [21], it gives us valuable information about which descriptors influence cellular uptake. By adding 20% noise around our calculated CP-response, we included the expected variability of the CP-responses, caused by experimental variations as well as by our assumptions. We evaluated the most robust descriptors, *i.e.* those descriptors which were incorporated in more than half of the obtained MLR-models. This MLR-analysis revealed that a positive charge, represented by the basic amino acids arginine and lysine, and an amphipathic structure are discriminating properties for cellular influx of peptides. We also identified the symmetry and the compactness of the peptide structure as determining. Furthermore, the 3D-MoRSE descriptors indicate that certain patterns in the molecular structure influence whether a peptide is efficiently cell-penetrating or not. This refers to an amphipathic structure or more in general to recurrent functional groups, like *e.g.* the guanidinium group of arginine. Indeed, based on the 3D-MoRSE descriptors, a favoured cellular influx is predicted for the amphipathic peptides. The results of the MLR-analysis correspond well with the identified important features for cellular uptake during the exploration of the chemical space of the 186 peptides.

**Figure 4:**
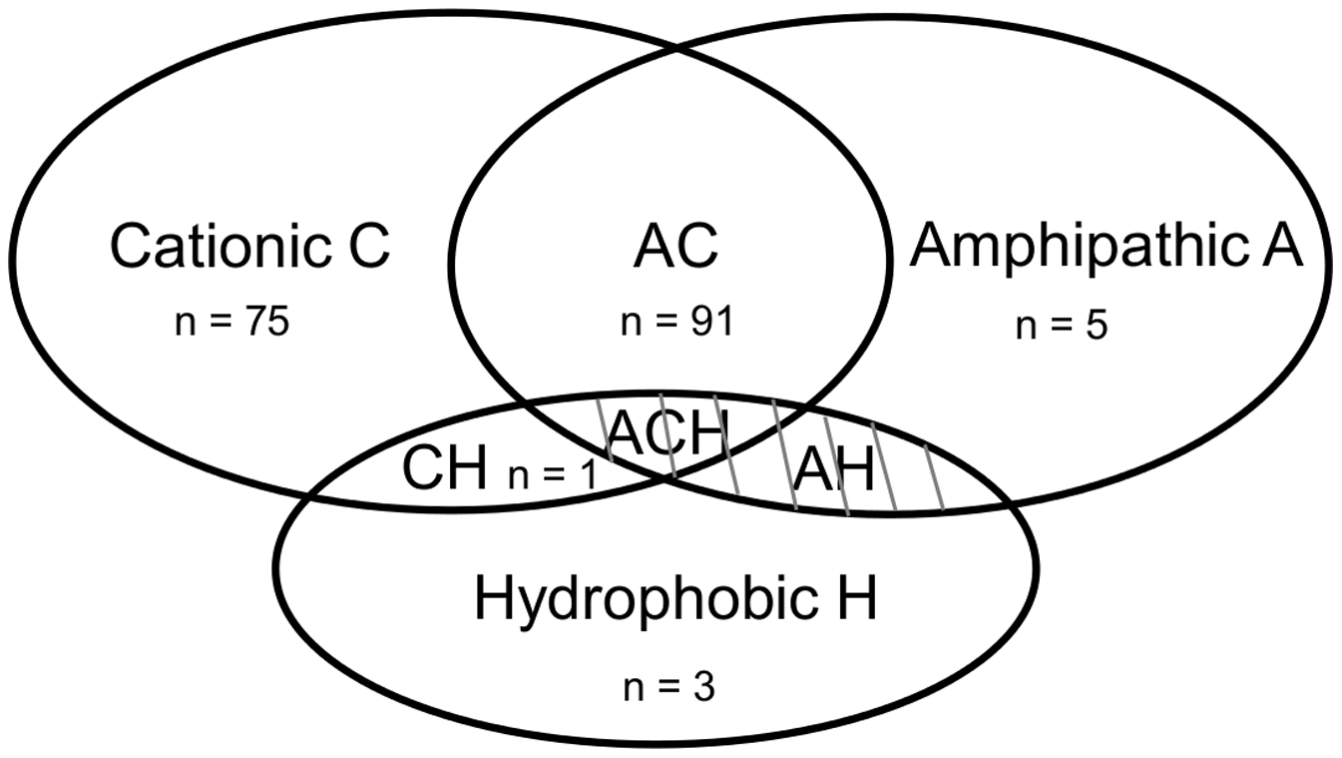
Schematic representation of the main CPP chemical classes from our dataset.

Cell-penetrating peptides form a chemically diverse group of peptides, as we demonstrated during the PCA-analysis, and can be classified in three chemically different groups according to Milletti [6]: *(1) cationic CPPs (C)*, which contain a stretch of positive charges and their 3D-structure is not an amphipathic helix. *(2) Amphipathic CPPs (A)*, which are characterized by a hydrophobic and hydrophilic part by adapting a helix structure. Amphipathic peptides may have a cationic nature (AC) or their hydrophilic part can be neutral, anionic or polar (A). The *(3) hydrophobic CPPs (H)* are peptides containing only apolar residues, with low net charge or have hydrophobic amino acid groups that are crucial for cellular uptake. Hydrophobic CPPs may also have a cationic (CH) or amphipathic nature (AC). In Table S3, the chemical classes of the individual peptides of our dataset are listed and are schematically visualized in Figure 4. Using this chemical classification method, there is a clear overlap demonstrated for the different classes, especially for the amphipathic-cationic peptides.

We believe that our CP-response, as a more objective and quantitative measure for cellular penetration, will foster the discussion of the cellular uptake mechanisms, as well as the definition and the classification of the CPPs.

### Conclusion

When gathering quantitative data for cellular influx of peptides, it was clear that harmonization of these studies is highly needed. By defining a cell-penetrating response, the quantitative evaluation of the cellular influx characteristics of 186 peptides was possible. This CP-response, together with chemo-molecular descriptors of the peptides, was used to explore the chemical-functional space of CPPs. Our study indicated that besides already reported CPP-determing features, like *i.a.* positive charge and amphipathicity, also the shape, complexity and compactness of the structures, play an import role for influx into the cell. As our CP-response is a more objective and quantitative measure for cellular penetration of peptides, it will help to classify these peptides, to unravel the different uptake mechanisms, as well as to establish a common evaluation tool.

## Supporting Information

Figure S1 **Score plot of the first versus the second principal component of the PCA-analysis of 186 peptides after dividing their descriptors by the molecular weight.** The colors of the clusters correspond with the clusters found in the score plot of the PCA-analysis using the original descriptors (Figure 2). For each cluster, some examples of peptides are indicated.
(PDF)

Table S1 **Overview of the 186 (non-) CPPs, including their CP-response.**
(PDF)

Table S2 **List of cell-penetrating peptides whose CP-response is an outlier.**
(PDF)

Table S3 **Classification of (non-) CPPs based on chemical class, literature data and their uptake mechanisms.**
(PDF)

Table S4 **Summary of the PCA-analysis of the descriptors divided by the molecular weight, describing the eigenvalues of the covariance matrix, the total variance explained (cumulative R^2^) and the predictive ability (cumulative Q^2^).**
(PDF)
