# Supplementary material for: Chemical-Functional Diversity in Cell-Penetrating Peptides
Source: PLoS One. 2013 Aug 9;8(8):e71752. doi: 10.1371/journal.pone.0071752 (PMC3739727; doi:10.1371/journal.pone.0071752)
Supplement: Figure S1 — Score plot of the first versus the second principal component of the PCA-analysis of 186 peptides after dividing their descriptors by the molecular weight. The colors of the clusters correspond with the clusters found in the score plot of the PCA-analysis using the original descriptors (Figure 2). For each cluster, some examples of peptides are indicated. (PDF) [file pone.0071752.s001.pdf]

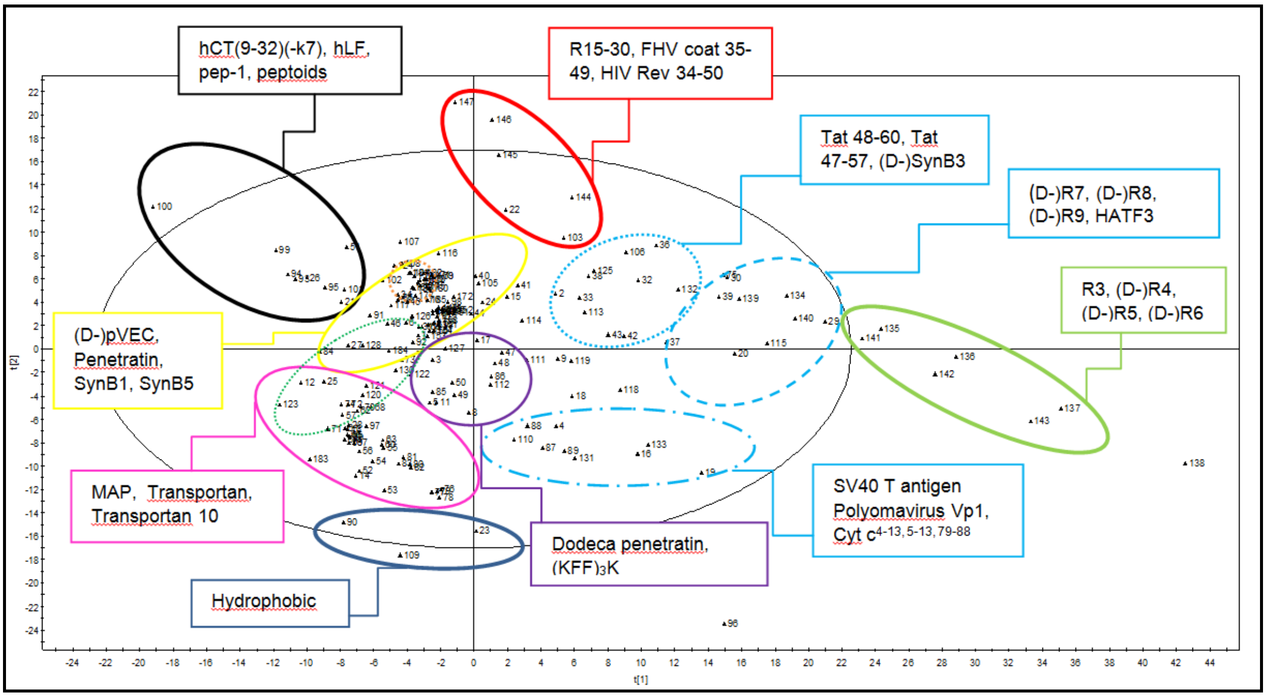

Figure S1. Score plot of the first versus the second principal component of the PCA-analysis of 186 peptides after dividing their descriptors by the molecular weight.

The colors of the clusters correspond with the clusters found in the score plot of the PCA-analysis using the original descriptors (Figure 2). For each cluster, some examples of peptides are indicated.
