# Supplementary material for: Chemical-Functional Diversity in Cell-Penetrating Peptides
Source: PLoS One. 2013 Aug 9;8(8):e71752. doi: 10.1371/journal.pone.0071752 (PMC3739727; doi:10.1371/journal.pone.0071752)
Supplement: Table S1 — Overview of the 186 (non-) CPPs, including their CP-response. (PDF) [file pone.0071752.s002.pdf]

Table S1. Overview of the 186 (non-) CPPs, including their CP-response.

Peptide ID	Peptide name	Sequence	Number of AA	CP-response	Ref.
1	pVEC	LLIILRRRIRKQAHAHSK	18	1.318	17, 25, 27, 53, 66
2	Tat 48-60	GRKKRRQRRRPPQ	13	0.221	17, 26, 34, 38, 66
3	APH-1 (85-98)	VFRFAYYKLLKKA	13	0.188	26
4	APH-1 (236-246)	LRSIQRSLLCK	11	0.039	26
5	Nicastrin (38-53)	RKIYIPLNKTAPCVR	15	0.345	26
6	Nicastrin (414-434)	RRPNQSQPLPPSSLQRFLRAR	21	0.016	26
7	Nicastrin (616-635)	RLPRCVRSTARLARALSPAF	20	0.031	26
8	Presenilin-1 (97-109)	VATIKSVSFYTRK	13	0.008	26
9	Presenilin-1 (305-317)	AQRRVSKNSKYNA	13	0.016	26
10	D-pVEC	lliilrrrirkqahahsk	18	0.824	7, 27, 53
11	$(KFF)_3K$	KFFKFFKFFK	10	1.837	27
12	*Transportan*	*GWTLNSAGYLLGKINLKALAALAKKIL*	*27*	*(4.357)*	*17, 28, 68*
13	Transportan 10	AGYLLGKINLKALAALAKKIL	21	1.641	28, 30, 32, 34, 66
14	MAP	KLALKLALKALKAALKLA	18	1.716	13, 17, 29, 37, 53
15	Syn B1	RGGRLSYSRRRFSTSTGR	18	0.063	17, 39
16	Polyomavirus Vp_1	APKRKSGVSK	10	0.028	17
17	Bac1-15	RRIRPRPPRLPRPRP	15	0.035	17
18	NF-κB	VQRKRQKLMP	10	0.025	17
19	SV40-T antigen	PKKKRKV	7	0.028	17
20	$HATF_3$	ERKKRRRE	8	0.023	17
21	hCT(9-32)	LGTYTQDFNKFHTFPQTAIGVGAP	24	0.024	17
22	HIV-1 Rev (34-50)	TRQARRNRRRRWRERQR	17	1.460	17, 38, 56
23	Integrin	VTVLALGALAGVGVG	15	0.549	17
24	DPV6	GRPRESGKKRKRKRLKP	17	0.073	17
25	$S4_{13}PV$	ALWKTLLKKVLKAPKKKRKV	20	0.781	17
26	Pep-1	KETWWETWWTEWSQPKKKRKV	21	0.105	17
27	MPG	GALFLGWLGAAGSTMGAWSQPKKKRKV	27	1.065	17
28	Poly-P (SAP)	VRLPPPVLRPPPVLRPPP	18	0.027	17
29	R7	RRRRRRR	7	0.395	12, 16, 17, 36
30	R9	RRRRRRRRR	9	1.000	17, 31, 33, 41, 42, 47, 63
31	pVEC scrambled	IAARIKLRSRQHIKLRHL	18	0.101	7, 17
32	Tat 47-57	YGRKKRRQRRR	11	0.309	30, 33, 42, 58
33	Tat 48-59	GRKKRRQRRRPP	12	0.192	31, 63
34	*M918*	*MVTVLFRRLRIRRACGPPRVRV*	*22*	*(7.158)*	*32*
35	Penetratin	RQIKIWFQNRRMKWKK	16	1.000	33, 39, 44
36	R11	RRRRRRRRRRR	11	0.211	34
37	RL9	RRLLRRLRR	9	0.134	35
38	RW9	RRWWRRWRR	9	1.301	35
39	R7W	RRRRRRRW	8	1.687	36
40	VP22	NAKTRRHERRRKLAIER	17	0.161	38
41	D-Syn B1	rggrlsysrrrfststgr	18	0.089	39
42	Syn B3	RRLSYSRRRF	10	0.126	39
43	D-Syn B3	rrlsysrrrf	10	0.185	39
44	Syn B5	RGGRLAYLRRRWAVLGR	17	1.159	39
45	ARF (1-22)	MVRRFLVTLRIRRACGPPRVRV	22	1.641	40
46	ARF (1-22) scrambled	FVTRGCPRRLVARLIRVMVPRR	22	1.296	40
47	ARF (2-14)	VRRFLVTLRIRRA	13	0.950	40
48	ARF (2-14) scrambled	RVRILARFLRTRV	13	0.173	40
49	ARF (19-31)	RVRVFVVHIPRLT	13	1.382	40
50	ARF (19-31) scrambled	VIRVHFRLPVRTV	13	0.259	40
51	hLF	KCFQWQRNMRKVRGPPVSCIKR *(disulfide 2-19)*	22	0.952	41
52	MAP (II)	KALAALLKKLAKLLAALK	18	0.181	13
53	MAP (III)	KLALKLALKALKAALK	16	0.115	13
54	MAP (VII)	KLALKLALKALQAALQLA	18	0.022	13
55	MAP (VIII)	KLALQLALQALQAALQLA	18	0.243	13

56	MAP (XI)	LKTLATALTKLAKTLTTL	18	0.177	13
57	MAP (XIII)	LKTLTETLKELTKTLTEL	18	0.052	13
58	MAP (XV)	RQIKIWFQNRRMKWKK	16	0.104	13
59	KLA 1	KLALKLALKAWKAALKLA	18	0.085	13
60	KLA 2	KLALKAALKAWKAAAKLA	18	0.008	13
61	KLA 11	KITLKLAIKAWKLALKAA	18	0.025	13
62	KLA 5	KIAAKSIAKIWKSILKIA	18	0.036	13
63	KLA 12	KALAKALAKLWKALAKAA	18	0.079	13
64	KLA 13	KLALKLALKWAKLALKAA	18	0.008	13
65	KLA 14	KLLAKAAKKWLLLALKAA	18	0.015	13
66	KLA 9	KLLAKAALKWLLKALKAA	18	0.023	13
67	KLA 10	KALKKLLAKWLAAAKALL	18	0.115	13
68	KLA 15	KLAAALLKKWKKLAAALL	18	0.120	13
69	KLA 8	KALAALLKKWAKLLAALK	18	0.181	13
70	TP10-1	AGYLLGPINLKALAALAKKIL	21	1.223	11
71	TP10-2	AGYLLGKINLKPLAALAKKIL	21	0.749	11
72	TP10-3	AAYLLAKINLKALAALAKKIL	21	1.243	11
73	TP10-4	AGYLLGKINLRALAALARRIL	21	1.263	11
74	TP10-5	AGYLLGKINLKKLAKL(Aib)KKIL[1]	21	2.141	11
75	D-R9	rrrrrrrrr	9	1.389	12, 43
76	MP	INLKALAALAKKIL	14	0.570	45
77	*iMP*	*inlkalaalakkil*	*14*	*(3.602)*	*45*
78	rMP	LIKKALAALAKLNI	14	0.352	45
79	riMP	likkalaalaklni	14	0.288	45
80	MitP	INLKKLAKL(Aib)KKIL[1]	14	2.712	45, 46
81	iMitP	inlkklakl(Aib)kkil	14	2.272	45
82	rMitP	LIKK(Aib)LKALKKLNI	14	0.531	45
83	riMitP	likk(Aib)lkalkklni[1]	14	0.837	45
84	Cyt c^{77-101}	GTKMIFVGIKKKEERADLIAYLKKA	25	0.179	46
85	Cyt c^{86-101}	KKKEERADLIAYLKKA	16	0.006	46
86	Cyt c^{79-92}	KMIFVGIKKKEERA	14	0.013	46
87	Cyt c^{79-88}	KMIFVGIKKK	10	0.006	46
88	Cyt c^{4-13}	EKGKKIFIMK	10	0.013	46
89	Cyt c^{5-13}	KGKKIFIMK	9	0.069	46
90	MTS	AAVALLPAVLLALLAP	16	0.315	48
91	*P14LRR*	*see fig. 1 reference 49*	-	*(7.958)*	*49*
92	P11LRR	see fig. 1 reference 49	-	1.623	49, 50
93	*$(P10LRR\text{-}Gly)_2$-C5*	*see fig. 2 reference 50*	-	*(84.034)*	*50*
94	*$(P10LRR\text{-}\beta\text{-}Ala)_2$-C5*	*see fig. 2 reference 50*	-	*(66.648)*	*50*
95	*$(P10LRR\text{-}ABUA)_2$-C5*	*see fig. 2 reference 50*	-	*(73.892)*	*50*
96	VPTLK	VPTLK	5	0.002	51
97	*MAP(Aib)*	*KL(Aib)LKL(Aib)LK(Aib)LKA(Aib)LKL(Aib)*[1]	*18*	*(125.240)*	*52*
98	sC18	GLRKRLRKFRNKIKEK	16	0.166	54
99	hCT(18-32)-k7	KFHTFPQTAIGVGAP KKRKAPKKKRKFA↵	28	0.183	54, 57
100	N-E5L-hCT(18-32)-k7	*GLLEALAELLE*KFHTFPQTAIGVGAP KKRKAPKKKRKFA↵	39	0.364	54
101	N-E5L-sC18	GLLEALAELLEGLRKRLRKFRNKIKEK	27	0.150	54
102	N-E5L-Tat 48-60	GLLEALAELLEGRKKRRQRRRPPQ	24	0.507	54
103	*FHV coat (35-49)*	*RRRRNRTRRNRRRVR*	*15*	*(4.127)*	*56*
104	*PasTat*	*FFLIPKGGRKKRRQRRRPPQ*	*20*	*(4.426)*	*55*
105	BMV Gag (7-25)	KMTRAQRRAAARRNRWTAR	19	0.288	56
106	HTLV-II Rex (4-16)	TRRQRTRRARRNR	13	0.288	56
107	Human cJun (252-279)	RIKAERKRMRNRIAASKSRKRKLERIAR	28	2.744	56
108	Human cFos (139-164)	KRRIRRERNKMAAAKSRNRRRELTDT	26	0.199	56
109	K-FGF (Kaposi fibroblast growth factor)	AAVLLPVLLAAP	12	0.200	58
110	PreS2-TLM (PreS2-derived translocatory motif)	PLSSIFSRIGDP	12	0.224	58
111	PTD4 (Protein transduction domain 4)	PIRRRKKLRRLK	12	0.658	58
112	α-peptide/β-peptoid chimera 2	see fig. 1 reference 59	-	0.185	59
113	α-peptide/β-peptoid chimera 6	see fig. 1 reference 59	-	2.040	59
114	α-peptide/β-peptoid chimera 8	see fig. 1 reference 59	-	2.102	59
115	$hArg_8$ ($Homoarginine_8$)	see fig. 1 reference 59	-	0.464	59
116	PenArg	RQIRIWFQNRRMRWRR	16	1.810	60
117	PenLys	KQIKIWFQNKKMKWKK	16	0.041	60

118	aca-[Lys(Nys$^+$)-aca]$_4$-Lys(Nys$^+$)	aca-[Lys(Nys$^+$)-aca]$_4$-Lys(Nys$^+$)[2]	-	0.001	61
119	aca-[Lys(Nys$^+$)-aca]$_6$-Lys(Nys$^+$)	aca-[Lys(Nys$_+$)-aca]$_6$-Lys(Nys$^+$)[2]	-	0.005	61
120	M511	FLGKKFKKYFLQLLK	15	0.599	62
121	M551	KGKFQLYLKLKFKFL	15	0.240	62
122	G53-1	IVIAKLKANLMCKTCRLAK	19	0.150	62
123	G53-2	AIGVNYLVKFIKVIAIVIAKLKA	23	0.839	62
124	*Kno ref. 63*<br>*(Knotted-1 homeodomain third helix)*	*KQIKINNWFINQRKRHWK*	*18*	*(7.358)*	*63*
125	R6/W3	RRWWRRWRR	9	0.910	63
126	Phe$^{6.14}$-penetratin	RQIKIFFQNRRMKFKK	16	0.727	64
127	Dodeca-penetratin	RQIKIWFRKWKK	12	1.154	64
128	MG2d (Magainin 2 analogue)	GIGKFLHSAKKWGKAFVGQIMNC	23	0.798	65
129	BF2d (Buforin 2 analogue)	TRSSRAGLQWPVGRVHRLLRKGGC	24	0.953	65
130	YTA2	YTAIAWVKAFIRKLRK	16	1.545	66
131	K9	KKKKKKKKK	9	0.119	12
132	H9	HHHHHHHHH	9	0.068	12
133	O9	OOOOOOOOO	9	0.068	12
134	R8	RRRRRRRR	8	0.639	12, 16
135	R6	RRRRRR	6	0.194	12, 16
136	R5	RRRRR	5	0.077	12, 16
137	R4	RRRR	4	0.074	12
138	R3	RRR	3	0.055	12
139	D-R8	rrrrrrrr	8	1.577	12
140	D-R7	rrrrrrr	7	1.071	12
141	D-R6	rrrrrr	6	0.323	12
142	D-R5	rrrrr	5	0.115	12
143	D-R4	rrrr	4	0.098	12
144	R15	RRRRRRRRRRRRRRR	15	1.345	12
145	R20	RRRRRRRRRRRRRRRRRRRR	20	0.764	12
146	R25	RRRRRRRRRRRRRRRRRRRRRRRRR	25	0.400	12
147	R30	RRRRRRRRRRRRRRRRRRRRRRRRRRRRRR	30	0.109	12
148	[Ala$_1$]pVEC	ALIILRRRIRKQAHAHSK	18	0.304	7
149	[Ala$_2$]pVEC	LAIILRRRIRKQAHAHSK	18	0.507	7
150	[Ala$_3$]pVEC	LLAILRRRIRKQAHAHSK	18	0.406	7
151	[Ala$_4$]pVEC	LLIALRRRIRKQAHAHSK	18	0.659	7
152	[Ala$_5$]pVEC	LLIIARRRIRKQAHAHSK	18	0.456	7
153	[Ala$_6$]pVEC	LLIILARRIRKQAHAHSK	18	2.433	7
154	[Ala$_7$]pVEC	LLIILRARIRKQAHAHSK	18	1.115	7
155	[Ala$_8$]pVEC	LLIILRRAIRKQAHAHSK	18	2.129	7
156	[Ala$_9$]pVEC	LLIILRRRARKQAHAHSK	18	0.760	7
157	[Ala$_{10}$]pVEC	LLIILRRRIAKQAHAHSK	18	1.470	7
158	[Ala$_{11}$]pVEC	LLIILRRRIRAQAHAHSK	18	1.774	7
159	[Ala$_{12}$]pVEC	LLIILRRRIRKAAHAHSK	18	1.420	7
160	[D-Ala$_{13}$]pVEC	LLIILRRRIRKQaHAHSK	18	1.825	7
161	[Ala$_{14}$]pVEC	LLIILRRRIRKQAAAHSK	18	0.558	7
162	[D-Ala$_{15}$]pVEC	LLIILRRRIRKQAHaHSK	18	1.115	7
163	[Ala$_{16}$]pVEC	LLIILRRRIRKQAHAASK	18	1.521	7
164	[Ala$_{17}$]pVEC	LLIILRRRIRKQAHAHAK	18	2.129	7
165	[Ala$_{18}$]pVEC	LLIILRRRIRKQAHAHSA	18	2.079	7
166	retro-pVEC	KSHAHAQKRIRRRLIILL	18	0.406	7
167	Ap1	AQIKIWFQNRRMKWKK	16	0.289	9
168	Ap2	RAIKIWFQNRRMKWKK	16	1.333	9
169	Ap3	RQAKIWFQNRRMKWKK	16	0.111	9
170	Ap4	RQIAIWFQNRRMKWKK	16	0.511	9
171	Ap5	RQIKAWFQNRRMKWKK	16	0.244	9
172	Ap6	RQIKIAFQNRRMKWKK	16	0.222	9
173	Ap7	RQIKIWAQNRRMKWKK	16	0.089	9
174	Ap8	RQIKIWFANRRMKWKK	16	0.778	9
175	Ap9	RQIKIWFQARRMKWKK	16	0.978	9
176	Ap10	RQIKIWFQNARMKWKK	16	0.400	9
177	Ap11	RQIKIWFQNRAMKWKK	16	0.242	9
178	Ap12	RQIKIWFQNRRAKWKK	16	0.644	9
179	Ap13	RQIKIWFQNRRMAWKK	16	0.378	9
180	Ap14	RQIKIWFQNRRMKAKK	16	0.356	9
181	Ap15	RQIKIWFQNRRMKWAK	16	0.411	9
182	Ap16	RQIKIWFQNRRMKWKA	16	0.444	9
183	SAP (E)	VELPPPVELPPPVELPPP	18	0.007	67

184	Eng (Engrailed-2 homeodomain third helix)	SQIKIWFQNKRAKIKK	16	1.235	69
185	Hox (HoxA-13 homeodomain third helix)	RQVTIWFQNRRVKEKK	16	0.500	69
186	*Kno ref. 69 (Knotted-1 homeodomain third helix)*	*KQINNWFINQRKRHWK*	*16*	*(11.118)*	*69*

Peptides whose unified response is an outlier are indicated in italic. Their unreliable CP-responses were not considered during data-evaluation.

[1]Aib = alpha amino-isobutyric acid

[2]Aca = $NH_2(CH_2)_5CO$-; Lys(Nys^+) = Redox amino acid = Lys with 1,4-dihydrotriognelline side chain
