# Supplementary material for: Chemical-Functional Diversity in Cell-Penetrating Peptides
Source: PLoS One. 2013 Aug 9;8(8):e71752. doi: 10.1371/journal.pone.0071752 (PMC3739727; doi:10.1371/journal.pone.0071752)
Supplement: Table S2 — List of cell-penetrating peptides whose CP-response is an outlier. (PDF) [file pone.0071752.s003.pdf]

Table S2. List of cell-penetrating peptides whose CP-response is an outlier.

Peptide ID	Peptide Name	Reference	Details
12	Transportan	17	In reference 17, transportan showed higher cell-specificity for HeLa cells, resulting in a higher response for cellular influx in comparison with other results for transportan.
34	M918	32	In the quantitative uptake assay, M918 showed a 7 times higher influx than penetratin.
91	P14LRR	49	P14LRR showed an about 20 times higher uptake than Tat 47-57, the positive control.
93	(P10LRR-Gly)$_2$-C5	50	The dimeric cationic amphiphilic polyproline helices showed an about 200 times higher uptake responses than the positive control Tat 47-57.
94	(P10LRR-β-Ala)$_2$-C5	50	
95	(P10LRR-ABUA)$_2$-C5	50	
97	MAP(Aib)	52	The internal positive control MAP showed a much lower influx response in the tested A549 cells (*cfr*. cell-specificity)
103	FHV Coat (35-49)	56	FHV coat (35-49) showed a 21 times higher cellular uptake than the positive control Tat 48-60.
104	PasTat	55	PasTat has a 20 times higher uptake than the positive control Tat.
124	Kno *(ref 63)*	63	Kno has a 7 times higher uptake than the positive control penetratin.
186	Kno *(ref 69)*	69	Kno has a 10 times higher uptake than the positive control penetratin.
