# Supplementary material for: Chemical-Functional Diversity in Cell-Penetrating Peptides
Source: PLoS One. 2013 Aug 9;8(8):e71752. doi: 10.1371/journal.pone.0071752 (PMC3739727; doi:10.1371/journal.pone.0071752)
Supplement: Table S3 — Classification of (non-) CPPs based on chemical class, literature data and their uptake mechanisms. (PDF) [file pone.0071752.s004.pdf]

Table S3. Classification of (non-) CPPs based on chemical class, literature data and their uptake mechanisms.

Peptide ID	Peptide name	Chemical class[1]	CP-response	Ref.	Literature evaluation[2]	Comment	Uptake mechanism[3]	Comment
1	pVEC	AC	1.318	17	High CPP	-	2	Unspecified
				25	CPP	≥ penetratin	1 and 2	Unsure
				27	CPP	> (KFF)$_3$K and penetratin	Not available	-
				53	CPP	≥ MAP, > penetratin	Not available	-
				66	CPP	= YTA2, > Tat 48-60, transportan 10, penetratin	1 and 2	Unspecified
2	Tat 48-60	C	0.221	17	Medium CPP	-	2	Unspecified
				26	CPP	< penetratin, APH (85-98), nicastrin (38-53)	2a	-
				34	CPP	= transportan 10, < penetratin < R11	2b	-
				38	CPP	> VP22, < HIV Rev (34-50) and penetratin	2a	-
				66	CPP	= transportan 10, > Tat 48-60, < pVEC and YTA2	1 and 2	Unspecified
3	APH-1 (85-98)	AU (AC)	0.188	26	CPP	> Tat 48-60, < penetratin and nicastrin (38-53)	2a	-
4	APH-1 (236-246)	AU (AC)	0.039	26	No CPP	-	Not applicable	-
5	Nicastrin (38-53)	AU (AC)	0.345	26	CPP	> Tat 48-60, < penetratin and APH (85-98)	2a	-
6	Nicastrin (414-434)	AU (C)	0.016	26	No CPP	-	Not applicable	-
7	Nicastrin (616-635)	AU (AC)	0.031	26	No CPP	-	Not applicable	-
8	Presenilin-1 (97-109)	AU (AC)	0.008	26	No CPP	-	Not applicable	-
9	Presenilin-1 (305-317)	AU (AC)	0.016	26	No CPP	-	Not applicable	-
10	D-pVEC	AC	0.824	7	CPP	≈ pVEC	2b > 2a	-
				27	CPP	≥ pVEC	Not available	-
				53	CPP	≥ pVEC	Not available	-
11	(KFF)$_3$K	AU (AC)	1.837	27	CPP	Less efficient than pVEC because of degradation	Not available	-
12	*Transportan*	AC	*(4.357)*	*17*	*High CPP*	-	*2*	*Unspecified*
				28	*CPP*	*> transportan 10*	*Not available*	-
				68	*CPP*	-	*1*	*Unspecified*
13	Transportan 10	AC	1.641	28	CPP	< transportan	Not available	-
				30	CPP	> penetratin >> Tat 47-57	Not available	-
				32	CPP	< M918	2a and 2b	-
				34	CPP	= Tat 48-60, < penetratin < R11	Not available	-
				66	CPP	= penetratin, > Tat 48-60, < pVEC and YTA2	1 and 2	*Unspecified*
14	MAP	AC	1.716	13	CPP	-	1 and 2	-
				17	High CPP	-	2	*Unspecified*
				29	CPP	-	1 and 2	Unspecified
				37	CPP	-	1 and 2	Unsure
				53	CPP	≥ pVEC, > penetratin	Not available	-
15	Syn B1	AC	0.063	17	Medium CPP	-	2	Unspecified
				39	CPP	-	2c	-
16	Polyomavirus Vp$_1$	AU (C)	0.028	17	Low CPP	Nearly unmeasurable uptake	2	Unspecified
17	Bac1-15	AU (C)	0.035	17	Medium CPP	-	2	Unspecified
18	NF-κB	C	0.025	17	Low CPP	Nearly unmeasurable uptake	2	Unspecified
19	SV40-T antigen	C	0.028	17	Low CPP	Nearly unmeasurable uptake	2	Unspecified
20	HATF$_3$	C	0.023	17	Low CPP	Nearly unmeasurable uptake	2	Unspecified
21	hCT(9-32)	A	0.024	17	Low CPP	Nearly unmeasurable uptake	2	Unspecified
22	HIV-1 Rev (34-50)	C	1.460	17	High CPP	-	2	Unspecified
				38	CPP	> penetratin > Tat 48-60 > VP22	2a	-
				56	CPP	-	Not available	-
23	Integrin	H	0.549	17	Medium CPP	-	2	Unspecified
24	DPV6	C	0.073	17	Medium CPP	-	2	Unspecified
25	S4$_{13}$PV	AC	0.781	17	High CPP	-	2	Unspecified
26	Pep-1	AC	0.105	17	Medium CPP	-	2	Unspecified
27	MPG	AC	1.065	17	Medium CPP	-	2	Unspecified

28	Poly-P (SAP)	AC	0.027	17	Low CPP	Nearly unmeasurable uptake	2	Unspecified
29	R7	C	0.395	12	CPP	> R6, < R8-9	3	-
				16	CPP	> R6, < R8-9	Not available	-
				17	High CPP	-	2	Unspecified
				36	CPP	< R7W	1 and 2	Unspecified, 2 = dominant mechanism
30	R9	C	1.000	17	High CPP	-	2	Unspecified
				31	CPP	-	Not available	-
				33	CPP	-	2	Unspecified
				35	High CPP	One of the most efficient known CPPs	1 and 2	Unspecified
				41	High CPP	Rapid	1e and 2	Unspecified
				42	CPP	-	1e, 2a and 2b	-
				43	CPP	-	1 < 2	Unspecified
				47	CPP	-	Not available	-
				63	CPP	-	Not available	-
31	pVEC scrambled	AU	0.101	7	Low CPP	<< pVEC , not efficient	2b > 2a	-
				17	Medium CPP	-	2	Unspecified
32	Tat 47-57	C	0.309	30	CPP	<< penetratin < transportan 10	1 and 2	Unspecified
				33	CPP	-	2	Unspecified
				42	CPP	-	1e, 2a and 2b	-
				58	CPP	> K-FGF and PreS2-TLM	Not available	-
33	Tat 48-59	C	0.192	31	CPP	< penetratin < R9	Not available	-
				63	CPP	-	Not available	-
34	*M918*	*AC*	*(7.158)*	*32*	*High CPP*	*Excellent novel CPP, > penetratin and transportan 10*	*2a*	-
35	Penetratin	C	1.000	17	High CPP	-	2	Unspecified
				29	CPP	-	2	Unspecified
				32	CPP	-	2a and 2b	-
				33	CPP	-	2	Unspecified
				39	CPP	-	2c	-
				42	CPP	-	1e, 2a and 2b	-
				44	CPP	-	1 and 2	Unspecified
				60	CPP	-	2a	-
				64	CPP	-	2b	-
36	R11	C	0.211	34	CPP	> penetratin > Tat 48-60, transportan 10	Not available	-
37	RL9	AC	0.134	35	Low CPP	Poor	1 and 2	Unspecified
38	RW9	AC	1.301	35	High CPP	One of the most efficient known CPPs	1 and 2	Unspecified
39	R7W	C	1.687	36	CPP	Efficient	1 and 2	Unspecified, 2 = dominant
40	VP22	AU (C)	0.161	38	CPP	< Tat 48-60 < penetratin < HIV Rev (34-50)	2a	-
41	D-Syn B1	AC	0.089	39	CPP	-	2c	-
42	Syn B3	AU (C)	0.126	39	CPP	-	2c	-
43	D-Syn B3	AU (C)	0.185	39	CPP	-	2c	-
44	Syn B5	AU (AC)	1.159	39	CPP	-	2c	-
45	ARF (1-22)	AC	1.641	40	High CPP	≈ transportan 10	2b	-
46	ARF (1-22) scrambled	AU	1.296	40	High CPP	≈ transportan 10	2b	-
47	ARF (2-14)	AC	0.950	40	High CPP	≈ transportan 10	Not available	-
48	ARF (2-14) scrambled	AU	0.173	40	Low CPP	-	Not available	-
49	ARF (19-31)	AC	1.382	40	High CPP	≈ transportan 10	Not available	-
50	ARF (19-31) scrambled	AU	0.259	40	Low CPP	-	Not available	-
51	hLF	AC	0.952	41	High CPP	Rapid	1e and 2	2 unspecified
52	MAP (II)	AC	0.181	13	CPP	-	1 and 2	Unspecified
53	MAP (III)	AC	0.115	13	CPP	≈ MAP	1 and 2	Unspecified
54	MAP (VII)	AC	0.022	13	Low CPP	Slow	1 and 2	Unspecified
55	MAP (VIII)	A	0.243	13	CPP	> MAP	1 and 2	Unspecified
56	MAP (XI)	AC	0.177	13	CPP	≈ MAP	1 and 2	Unspecified
57	MAP (XIII)	A	0.052	13	CPP	≈ MAP	1 and 2	Unspecified
58	MAP (XV)	AC	0.104	13	CPP	≈ MAP	1 and 2	Unspecified
59	KLA 1	AC	0.085	13	CPP	-	1 and 2	Unspecified
60	KLA 2	AC	0.008	13	Low CPP	Slow	1 and 2	Unspecified
61	KLA 11	AC	0.025	13	Low CPP	Slow	1 and 2	Unspecified
62	KLA 5	AC	0.036	13	Low CPP	Slow	1 and 2	Unspecified
63	KLA 12	AC	0.079	13	CPP	-	1 and 2	Unspecified
64	KLA 13	AC	0.008	13	Low CPP	Slow	1 and 2	Unspecified
65	KLA 14	AC	0.015	13	Low CPP	Slow	1 and 2	Unspecified
66	KLA 9	AC	0.023	13	Low CPP	Slow	1 and 2	Unspecified
67	KLA 10	AC	0.115	13	CPP	-	1 and 2	Unspecified
68	KLA 15	AC	0.120	13	CPP	-	1 and 2	Unspecified
69	KLA 8	AC	0.181	13	CPP	-	1 and 2	Unspecified
70	TP10-1	AC	1.223	11	CPP	< transportan 10	1d	-
71	TP10-2	AC	0.749	11	CPP	< transportan 10	1d	-
72	TP10-3	AC	1.243	11	CPP	< transportan 10	1d	-

73	TP10-4	AC	1.263	11	CPP	< transportan 10	1d	-
74	TP10-5	AC	2.141	11	CPP	> transportan 10	1d	-
75	D-R9	C	1.389	12	CPP	Effective, (> (D-)R6-8)	3	-
				43	CPP	< R9	1 > 2	Unspecified
76	MP	AC	0.570	45	Low CPP	Low efficient	1	Unspecified
77	*iMP*	*AC*	*(3.602)*	*45*	*High CPP*	*Highly efficient, (> MitP, penetratin*	*1*	*Unspecified*
78	rMP	AC	0.352	45	Low CPP	Low efficient	Not applicable	-
79	riMP	AC	0.288	45	Low CPP	Low efficient	Not applicable	-
80	MitP	AC	2.712	45	Medium CPP	Efficient	1	Unspecified
				46	CPP	-	Not available	-
81	iMitP	AC	2.272	45	CPP	Strong propensity for cell penetration	Not available	-
82	rMitP	AC	0.531	45	Low CPP	Low efficient	Not available	-
83	riMitP	AC	0.837	45	Low CPP	Low efficient	Not available	-
84	Cyt c$^{77-101}$	AU (C)	0.179	46	High CPP	Extremely/very efficient	Not available	-
85	Cyt c$^{86-101}$	AU (C)	0.006	46	CPP	-	Not available	-
86	Cyt c$^{79-92}$	AU (C)	0.013	46	Not available	-	Not available	-
87	Cyt c$^{79-88}$	AU (C)	0.006	46	Not available	-	Not available	-
88	Cyt c$^{4-13}$	AU (C)	0.013	46	Not available	-	Not available	-
89	Cyt c$^{5-13}$	AU (C)	0.069	46	Not available	-	Not available	-
90	MTS	H	0.315	48	CPP	-	Not available	-
91	*P14LRR*	*AC*	*(7.958)*	*49*	*High CPP*	*Highly efficient class of CPPs*	*1*	*Unspecified*
92	P11LRR	AC	1.623	49	High CPP	Highly efficient class of CPPs	1 and 2	Unspecified
				50	CPP	-	1 and 2	Unspecified
93	*(P10LRR-Gly)$_2$-C5*	*AC*	*(84.034)*	*50*	*CPP*	*Uptake far superior to P11LRR and Tat 47-57*	*1 and 2*	*Unspecified*
94	*(P10LRR-β-Ala)$_2$-C5*	*AC*	*(66.648)*	*50*	*CPP*	*Uptake far superior to P11LRR and Tat 47-57*	*1 and 2*	*Unspecified*
95	*(P10LRR-ABUA)$_2$-C5*	*AC*	*(73.892)*	*50*	*CPP*	*Uptake far superior to P11LRR and Tat 47-57*	*1 and 2*	*Unspecified*
96	VPTLK	CH	0.002	51	CPP	-	Unknown	-
97	*MAP(Aib)*	*AC*	*(125.240)*	*52*	*High CPP*	-	*Not available*	-
98	sC18	AC	0.166	54	High CPP	Highly efficient	1 and 2	Unspecified
99	hCT(18-32)-k7	AU	0.183	54	CPP	-	2	Unspecified
				57	Medium CPP	Effective	Not available	-
100	N-E5L-hCT(18-32)-k7	AU	0.364	54	CPP	Faster and efficienter than hCT(18-32)-k7	Unclear	-
101	N-E5L-sC18	AC	0.150	54	CPP	Faster and efficienter than sC18	Unclear	-
102	N-E5L-Tat 48-60	AU (C)	0.507	54	CPP	Faster and efficienter than Tat 48-60	Unclear	-
103	*FHV coat (35-49)*	*C*	*(4.127)*	*56*	*High CPP*	*High efficient CPP and superior cellular uptake >Tat 48-60*	*2a*	-
104	*PasTat*	*C*	*(4.426)*	*55*	*CPP*	-	*Not available*	-
105	BMV Gag (7-25)	C	0.288	56	CPP	-	Not available	-
106	HTLV-II Rex (4-16)	C	0.288	56	CPP	-	Not available	-
107	Human cJun (252-279)	C	2.744	56	CPP	-	Not available	-
108	Human cFos (139-164)	C	0.199	56	CPP	-	Not available	-
109	K-FGF (Kaposi fibroblast growth factor)	H	0.200	58	CPP	< arginine-rich peptides: Tat 47-57, penetratin and PTD4	Not available	-
110	PreS2-TLM (PreS2-derived translocatory motif)	A	0.224	58	CPP	< arginine-rich peptides: Tat 47-57, penetratin and PTD4	Not available	-
111	PTD4 (Protein transduction domain 4)	C	0.658	58	CPP	> peptides: K-FGF and PreS2-TLM	Not available	-
112	α-peptide/β-peptoid chimera 2	AU	0.185	59	Medium CPP	Possesses efficient cellular uptake properties superior to well-described CPPs, ≈ Tat 47-57	1 and 2	Unspecified
113	α-peptide/β-peptoid chimera 6	AU	2.040	59	High CPP	Possesses efficient cellular uptake properties superior to well-described CPPs, > Tat 47-57	1 and 2	Unspecified
114	α-peptide/β-peptoid chimera 8	AU	2.102	59	High CPP	Possesses efficient cellular uptake properties superior to well-described CPPs , > Tat 47-57	1 and 2	Unspecified
115	hArg$_8$ (Homoarginine$_8$)	C	0.464	59	Medium CPP	Possesses efficient cellular uptake properties superior to well-described CPPs, ≈	1 and 2	Unspecified

						Tat 47-57		
116	PenArg	C	1.810	60	CPP	> penetratin > PenLys	2a	-
117	PenLys	C	0.041	60	CPP	< penetratin < PenArg	2a	-
118	aca-[Lys(Nys$^+$)-aca]$_4$-Lys(Nys$^+$)	AU	0.001	61	No CPP	-	Not applicable	-
119	aca-[Lys(Nys$^+$)-aca]$_6$-Lys(Nys$^+$)	AU	0.005	61	Low CPP	-	Not available	-
120	M511	AU (AC)	0.599	62	CPP	-	Not available	-
121	M551	AU (C)	0.240	62	CPP	-	Not available	-
122	G53-1	AU (AC)	0.150	62	Low CPP	-	Not available	-
123	G53-2	AU (AC)	0.839	62	CPP	-	Not available	-
124	*Kno ref. 61 (Knotted-1 homeodomain third helix)*	*C*	*(7.358)*	*63*	*CPP*	-	*Not available*	-
125	R6/W3	AC	0.910	63	CPP	-	Not available	-
126	Phe$^{6.14}$-penetratin	AU (C)	0.727	64	CPP	< penetratin	2b	-
127	Dodeca-penetratin	AU (AC)	1.154	64	CPP	≈ penetratin	2b	-
128	MG2d (Magainin 2 analogue)	AC	0.798	65	CPP	-	1b	-
129	BF2d (Buforin 2 analogue)	AC	0.953	65	CPP	-	Not available	-
130	YTA2	AC	1.545	66	CPP	= pVEC, > Tat 48-60, transportan 10, penetratin	1 and 2	Unspecified
131	K9	C	0.119	12	Low CPP	Not effective	Not available	-
132	H9	C	0.068	12	Low CPP	Not effective	Not available	-
133	O9	C	0.068	12	Low CPP	Not effective	Not available	-
134	R8	C	0.639	12	Medium CPP	Effective CPP (> R6-7, < R9)	3	-
				16	CPP	>R6-7, < R9	Not available	-
135	R6	C	0.194	12	Medium CPP	Effective CPP, < R7-9	3	-
				16	CPP	< R7-9	Not available	-
136	R5	C	0.077	12	Low CPP	Not effective CPP	Not available	-
				16	No CPP	-	Not applicable	-
137	R4	C	0.074	12	Low CPP	Not effective CPP	Not available	-
138	R3	C	0.055	12	Low CPP	Not effective CPP	Not available	-
139	D-R8	C	1.577	12	Medium CPP	Effective CPP, > (D-)R6-7, < (D-) R9	3	-
140	D-R7	C	1.071	12	Medium CPP	Effective CPP, > (D-)R6, < (D-) R8-9	3	-
141	D-R6	C	0.323	12	Medium CPP	Effective CPP, < (D-)R7-9	3	-
142	D-R5	C	0.115	12	Low CPP	Not effective CPP	Not available	-
143	D-R4	C	0.098	12	Low CPP	Not effective CPP	Not available	-
144	R15	C	1.345	12	Medium CPP	Effective CPP, > R6-9, but toxic	3	-
145	R20	C	0.764	12	Medium CPP	Effective CPP, < R9, R15, but toxic	3	-
146	R25	C	0.400	12	Low CPP	Not effective CPP, < R7, R9, R15, but toxic	Not available	-
147	R30	C	0.109	12	Low CPP	Not effective CPP	Not available	-
148	[Ala$_1$]pVEC	AC	0.304	7	CPP	Significant < pVEC	2b > 2a	-
149	[Ala$_2$]pVEC	AC	0.507	7	CPP	Significant < pVEC	2b > 2a	-
150	[Ala$_3$]pVEC	AC	0.406	7	CPP	Significant < pVEC	2b > 2a	-
151	[Ala$_4$]pVEC	AC	0.659	7	CPP	Significant < pVEC	2b > 2a	-
152	[Ala$_5$]pVEC	AC	0.456	7	CPP	Significant < pVEC	2b > 2a	-
153	[Ala$_6$]pVEC	AC	2.433	7	CPP	Significant > pVEC	2b > 2a	-
154	[Ala$_7$]pVEC	AC	1.115	7	CPP	≈ pVEC	2b > 2a	-
155	[Ala$_8$]pVEC	AC	2.129	7	CPP	Significant > pVEC	2b > 2a	-
156	[Ala$_9$]pVEC	AC	0.760	7	CPP	Significant > pVEC	2b > 2a	-
157	[Ala$_{10}$]pVEC	AC	1.470	7	CPP	≈ pVEC	2b > 2a	-
158	[Ala$_{11}$]pVEC	AC	1.774	7	CPP	≈ pVEC	2b > 2a	-
159	[Ala$_{12}$]pVEC	AC	1.420	7	CPP	≈ pVEC	2b > 2a	-
160	[D-Ala$_{13}$]pVEC	AC	1.825	7	CPP	≈ pVEC	2b > 2a	-
161	[Ala$_{14}$]pVEC	AC	0.558	7	CPP	Significant > pVEC	2b > 2a	-
162	[D-Ala$_{15}$]pVEC	AC	1.115	7	CPP	≈ pVEC	2b > 2a	-
163	[Ala$_{16}$]pVEC	AC	1.521	7	CPP	≈ pVEC	2b > 2a	-
164	[Ala$_{17}$]pVEC	AC	2.129	7	CPP	Significant > pVEC	2b > 2a	-
165	[Ala$_{18}$]pVEC	AC	2.079	7	CPP	≈ pVEC	2b > 2a	-
166	retro-pVEC	AC	0.406	7	CPP	Significant > pVEC	2b > 2a	-
167	Ap1	C	0.289	9	Low CPP	< penetratin	Unclear	-
168	Ap2	C	1.333	9	CPP	> penetratin	Unclear	-
169	Ap3	C	0.111	9	Low CPP	< penetratin	Unclear	-
170	Ap4	C	0.511	9	Low CPP	< penetratin	Unclear	-
171	Ap5	C	0.244	9	Low CPP	< penetratin	Unclear	-
172	Ap6	C	0.222	9	Low CPP	< penetratin	Unclear	-
173	Ap7	C	0.089	9	Low CPP	< penetratin	Unclear	-
174	Ap8	C	0.778	9	CPP	≈ penetratin	Unclear	-
175	Ap9	C	0.978	9	CPP	≈ penetratin	Unclear	-
176	Ap10	AC	0.400	9	Low CPP	< penetratin	Unclear	-

177	Ap11	C	0.242	9	Low CPP	< penetratin	Unclear	-
178	Ap12	C	0.644	9	CPP	< penetratin	Unclear	-
179	Ap13	C	0.378	9	Low CPP	< penetratin	Unclear	-
180	Ap14	C	0.356	9	Low CPP	< penetratin	Unclear	-
181	Ap15	C	0.411	9	Low CPP	< penetratin	Unclear	-
182	Ap16	C	0.444	9	Low CPP	< penetratin	Unclear	-
183	SAP (E)	A	0.007	67	Low CPP	Not highly efficient	2b	-
184	Eng<br>(Engrailed-2 homeodomain third helix)	C	1.235	69	CPP	-	Not available	-
185	Hox<br>(HoxA-13 homeodomain third helix)	C	0.500	69	CPP	-	Not available	-
186	*Kno ref. 67*<br>*(Knotted-1 homeodomain third helix)*	*C*	*(11.118)*	*69*	*High CPP*	*High uptake efficiency*	*Not available*	-

Peptides whose unified response is an outlier are indicated in italic. Their unreliable CP-responses were not considered during data-evaluation.

[1]Chemical classes of CPPs: A = amphipathic, C = cationic, H = hydrophobic, AC = intersection amphipathic-cationic, CH = intersection cationic-hydrophobic and AU = author unclassified. If AU, the recommended chemical class, according to ref. 6, is indicated between brackets.

[2]Literature evaluation = how the authors of the study estimated the cellular influx capacity of the investigated peptides: no, low, medium or high CPP. CPP alone means not specified by the authors.

[3]1 = direct penetration with 1a = inverted micelles, 1b = pore formation, 1c = carpet like model, 1d = membrane thinning and 1e = uptake through nucleation zones; 2 = endocytosis with 2a = micropinocytosis, 2b = endocytosis dependent on coat proteins (clathrin, caveolae or lipid raft mediated) and 2c = endocytosis independent on coat proteins; 3 = energy-dependent mechanism, but not endocytosis [according to ref. 2,12,72,73]
