# Supplementary material for: Chemical-Functional Diversity in Cell-Penetrating Peptides
Source: PLoS One. 2013 Aug 9;8(8):e71752. doi: 10.1371/journal.pone.0071752 (PMC3739727; doi:10.1371/journal.pone.0071752)
Supplement: Table S4 — Summary of the PCA-analysis of the descriptors divided by the molecular weight, describing the eigenvalues of the covariance matrix, the total variance explained (cumulative R2) and the predictive ability (cumulative Q2). (PDF) [file pone.0071752.s005.pdf]

Table S4. Summary of the PCA-analysis of the descriptors divided by the molecular weight, describing the eigenvalues of the covariance matrix, the total variance explained (cumulative R^2) and the predictive ability (cumulative Q^2).

Principal Component	Eigenvalue	Cumulative R^2	Cumulative Q^2
1	73.7	0.396	0.371
2	41.8	0.621	0.596
3	12.7	0.689	0.655
4	8.28	0.734	0.678
5	7.95	0.777	0.715
6	6.27	0.810	0.749
7	4.44	0.834	0.771
8	3.18	0.851	0.781
9	2.75	0.866	0.792
10	2.53	0.880	0.803
11	2.08	0.891	0.807
12	1.62	0.900	0.803
13	1.44	0.907	0.807
